# Improving Care for Patients Living with Prolonged Incurable Cancer

**DOI:** 10.3390/cancers13112555

**Published:** 2021-05-23

**Authors:** Mariken E. Stegmann, Olaf P. Geerse, Lia van Zuylen, Larissa Nekhlyudov, Daan Brandenbarg

**Affiliations:** 1 Department of General Practice & Elderly Care Medicine, University Medical Center Groningen, University of Groningen, 9700 CC Groningen, The Netherlands; m.e.stegmann@umcg.nl (M.E.S.); d.brandenbarg@umcg.nl (D.B.); 2 Academic Medical Center, Department of Pulmonary Medicine, Amsterdam University Medical Center, University of Amsterdam, 1105 AZ Amsterdam, The Netherlands; 3 Amsterdam University Medical Center, Department of Medical Oncology, 1105 AZ Amsterdam, The Netherlands; c.vanzuylen@amsterdamumc.nl; 4 Brigham and Women’s Hospital, Harvard Medical School, Boston, MA 02115, USA; Larissa_Nekhlyudov@dfci.harvard.edu

**Keywords:** palliative care, survivorship, primary care, care coordination

## Abstract

**Simple Summary:**

Not all patient with cancer can be cured. However, some patients with incurable cancer may expect to live for a substantial period of time. The number of patients in this group is increasing. These patients with ‘prolonged incurable cancer’ are often overlooked in research and clinical practice. They may have questions related to palliative care (e.g., about the end of life) and related to survivorship care (e.g., about late treatment effects). By itself, a palliative or survivorship perspective may therefore be insufficient to cover the wide range of physical and psychosocial problems that patients with prolonged incurable cancer encounter. Elements from both fields should therefore be delivered concordantly. This proposed new care model can further optimize care pathways for these patients. Furthermore, enhanced clinical awareness for this patient population as well as further research are urgently needed.

**Abstract:**

The number of patients that can no longer be cured but may expect to live with their cancer diagnosis for a substantial period is increasing. These patients with ‘prolonged incurable cancer’ are often overlooked in research and clinical practice. Patients encounter problems that are traditionally seen from a palliative or survivorship perspective but this may be insufficient to cover the wide range of physical and psychosocial problems that patients with prolonged incurable cancer may encounter. Elements from both fields should, therefore, be delivered concordantly to further optimize care pathways for these patients. Furthermore, to ensure future high-quality care for this important patient population, enhanced clinical awareness, as well as further research, are urgently needed.

## 1. Prolonged Incurable Cancer: Definition and Unique Needs

The number of patients that can no longer be cured but may be expected to live with their cancer diagnosis for a substantial period is increasing. Since the introduction of recent therapeutic advances, the period between a diagnosis of non-curative cancer and the end of life has rapidly increased for many solid malignancies [1,2,3]. For patients diagnosed with colorectal cancer with multiple metastases, the five-year survival rate is now approaching 14 percent [4]. For patients diagnosed with a high degree of microsatellite instability, immunotherapy is expected to significantly increase the five-year survival [5]. For metastatic breast and prostate cancer, treatment options are also expanding and the three-year survival rate is now approaching 50 percent [6]. The increasing use of immunotherapy across the cancer spectrum is likely to further increase this survival period for a multitude of patients [3,7,8]. As a result of this prolonged survival, the traditional dichotomy between patients with curable cancer and patients with incurable cancer who receive palliative or end-of-life care no longer suffices. In addition, the heterogeneity of this group of survivors with “prolonged incurable cancer” is expanding [9]. It may be comprised of patients who have completed initial therapy for metastatic disease, but continue to receive active treatment (e.g., targeted therapy or hormonal therapy for metastatic prostate cancer). Moreover, the group consists of patients with advanced cancer who do not receive therapy anymore and are subject to close follow-up and of patients with incurable cancer such as chronic lymphocytic leukemia who may not need treatment (until the time comes) [10].

Despite the increasing size and heterogeneity of this population, these patients have been largely overlooked, as a group, in current oncological guidelines [11,12]. This might partly be due to the lack of a consistent term to refer to this group of patients, with resultant confusion in the development of clinical programs and research interventions [13]. Earlier reports have suggested using the terms “metastatic cancer”, “stable or chronic cancer” or “patients in-between” [10,14,15]. However, as some metastases (e.g., a solitary metastasis in the liver or stage IV Hodgkin lymphoma) can be treated curatively, the term metastatic cancer does not seem to suffice. More importantly, patients may dislike terminology like chronic or stable cancer to describe their disease [16,17]. In this paper, we use the term “prolonged incurable cancer”, as suggested by others [18].

Attributing a term like “prolonged incurable cancer” is important because the problems these patients face are unique: they both experience problems associated with living with an incurable disease (e.g., palliative care), but due to their prolonged survival, they also face issues that may traditionally be related to survivorship [10]. In this perspective, we aim to describe the extent to which patients with prolonged incurable cancer can be treated and viewed from a palliative as well as a survivorship perspective. We briefly outline which problems these patients can experience by specifically focusing on both the physical and psychosocial domains. We conclude by providing strategies to optimize care pathways for patients with prolonged incurable cancer and outline important areas for future research.

## 2. Palliative Care

Palliative care is defined as an approach that sets out to improve the quality of life of patients and their caregivers who are faced with a life-limiting illness [19,20]. This is primarily done through early identification of eligible patients with recognition, prevention and treatment of (potential) physical, psychological, social or spiritual problems [20]. In recent years, a plethora of evidence has shown that early palliative care can effectively improve quality of life, reduce anxiety and depression, prolong survival and reduce the cost of care while improving its quality near to the end of life [21,22,23,24]. To facilitate early recognition, various tools and/or models have been suggested. One of these, the “surprise question”, has been extensively studied across a multitude of patient populations, although it has primarily been used in oncological settings [25,26]. The question “Would I be surprised if this patient dies in the next twelve months?” has been shown to adequately identify patients that are eligible for palliative care, and is, therefore, increasingly used [25]. However, this method may neglect the increasing number of patients diagnosed and living with prolonged incurable cancer, although they do suffer from a life-limiting illness and could benefit from particular aspects of palliative care [27,28]. Palliative care that comprises symptom management with a focus on the physical, psychological, social and spiritual domains should, therefore, be offered shortly after the diagnosis.

## 3. Survivorship Care

Patients with prolonged incurable cancer can also experience questions regarding survivorship care. Survivorship care consists of three core elements: (1) prevention and surveillance for recurrences and new cancers; (2) surveillance and management of physical and psychosocial long-term treatment effects; (3) care for general health: chronic disease management, health promotion and disease prevention [9,29,30]. One measure to convey and integrate such information is through a survivorship care plan. Traditionally, such plans are outlined once patients complete curative treatment of their cancer. However, also for patients with prolonged incurable cancer, these issues may become relevant and may negatively impact their quality of life. Next, we briefly outline which problems can be expected for patients with prolonged incurable cancer, both from the palliative and survivorship care perspectives.

## 4. Physical Problems

Patients with prolonged incurable cancer can experience symptoms of their cancer itself (e.g., pain) and/or have questions about the management of possible future symptoms (e.g., dyspnea close to the end of life). Furthermore, they can both experience acute and ongoing side-effects of current treatments (e.g., hot flashes due to continued hormonal therapy), as well as long-term and late effects of former treatments (e.g., complications of surgery, neurotoxicity of chemotherapy, hypothyroidism after thyroiditis due to immunotherapy) which may negatively impact their experienced quality of life [31,32,33]. For example, fatigue is described as a frequently encountered physical symptom after cancer [32], but it is currently unknown to what extent patients with prolonged incurable cancer suffer from this condition and whether evidence-based interventions are effective for this population of patients.

## 5. Psychosocial Problems

Cancer survivors are known to have higher levels of anxiety and depression compared to the reference population [34,35]. Yet, it is not known to what extent patients with prolonged incurable cancer suffer from these psychological symptoms. In the social domain, patients may experience problems with finding a new position in their relationships (e.g., partner, children, family and friends) after having assumed “the patient role” for a period of time [36]. Returning to work remains a troublesome issue, both due to physical effects like fatigue and cognitive problems and through uncertainty about life expectancy and doubts about the relative importance or desirability of continuing with a job [37,38,39]. Yet, for a large proportion of patients, returning to work is a financial necessity, which emphasizes the need to address this issue. This will be all the more relevant when living with a prolonged incurable disease. Such existential questions may also stretch far beyond issues within the social domain and encompass the meaning of life in general and related spiritual questions. Furthermore, patients with prolonged incurable cancer might experience uncertainty about their future or worry about what will happen to their loved ones if their disease worsens and causes deterioration or eventually death.

## 6. Improving Clinical Practice

**Figure 1:**
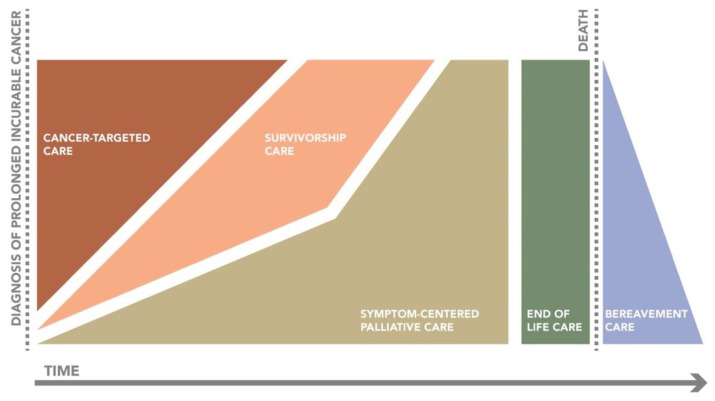
Proposed care model for patients with prolonged incurable cancer. Model adapted from original model by Lynn & Adamson [40]. Within this model, cancer-targeted care focuses on treatment of the tumor and/or metastases. Survivorship care is characterized by surveillance (for disease flare-ups), management of long-term physical and/or psychosocial symptoms, late treatment effects and general healthcare. Symptom-centered palliative care addresses acute or other physical and/or psychological symptoms as well as problems that may arise from the social or existential domain.

Due to the heterogeneity of the group of patients with prolonged incurable cancer, it is difficult to provide clear recommendations on how to best organize supportive care for this patient population. However, it is clear that the proportion of this group of patients is often underestimated and not always timely or adequately identified. As such, creating awareness and recognition for this particular subgroup of patients is a first and important step. All treating healthcare providers must identify which of their patients have prolonged incurable cancer and assess the physical and psychosocial symptoms and needs of these patients and manage them appropriately. Elements from both palliative care and survivorship care, as described above, should be delivered concordantly to all patients with prolonged incurable cancer. A visualization of this provided care model is displayed in Figure 1, as adapted from the original model by Lynn and Adamson [40].

Since a significant proportion of patients with prolonged incurable cancer will not visit the hospital frequently (e.g., either at intervals of three or six months), primary care providers would likely have to be the central point of contact for the majority of patients. This recommendation is in line with recent literature describing how patients with incurable cancer prefer to discuss issues pertaining to psychosocial or end-of-life care with their primary care providers [41]. Furthermore, in addition to focusing on physical and psychosocial problems, primary care providers are, as generalists, best suited to taking care of patients’ general health as the third core element of survivorship care. Because of the rapid development of new therapies and specific side-effects that may be related to the advancements in recent therapies, close collaboration with secondary care providers (e.g., medical oncologists or radiation therapists) is critical for clinicians working in primary care. In addition, the development of programs specially designed for this cross-section of palliative and survivorship care may be further promoted.

## 7. Research Agenda

Although the body of evidence for oncology patients focusing on care optimization, symptom management, palliative care and survivorship care is rapidly increasing, research specifically targeting this particular population of patients with prolonged incurable cancer lags behind. In line with previous research [10], we identify the following areas of research as highly important:

1. What is the epidemiology of patients living with prolonged incurable cancer (prevalence, demographic characteristics and tumor types)?
2. How and by whom can patients with prolonged incurable cancer be identified? Are there standardized methods by which this may be done (via medical record review, tumor registries, questionnaires, etc.)?
3. What is the epidemiology of experienced physical symptoms for patients with prolonged incurable cancer? How can they best be managed?
4. What is the epidemiology of experienced psychosocial symptoms for patients with prolonged incurable cancer? How can they best be managed?
5. How are care pathways for patients with prolonged incurable cancer currently organized and how can these be optimized? What are the roles for different healthcare providers, including primary care (e.g., general practitioner), in the care and care coordination for patients with prolonged incurable cancer?

## 8. Conclusions

Patients with prolonged incurable cancer are living with an illness with an unknown life expectancy. Both the volume and the heterogeneity of this patient population will be likely to increase in the years to come. Yet, not all problems that this unique patient population encounters are well-known or studied, and efforts should therefore be made to adequately recognize and alleviate them. Application of elements from the field of survivorship care and palliative care are of help and may be further optimized. To continually ensure future high-quality care for this important patient population, enhanced clinical awareness as well as further research are urgently needed.

## Data Availability

This perspective article does not report any new data.
